# Association of Dietary Approaches to Stop Hypertension Diet With the Risk of Osteoporosis and Fracture: A Systematic Review and Meta‐Analysis

**DOI:** 10.1002/fsn3.71892

**Published:** 2026-05-14

**Authors:** Bin Yang, Jiaojiao Hou, Xin Song, Sirui Zheng, Qin Deng, Rong Xiang, Xunying Zhao, Yang Qu, Linna Sha, Jiangbo Zhu, Bowen Lei, Ting Yu, Tao Han, Jinyu Zhou, Ting Liu, Maoyao Xia, Yangdan Zhong, Xiaobing Pu, Xia Jiang

**Affiliations:** ^1^ Department of Nutrition and Food Hygiene, West China School of Public Health and West China Fourth Hospital Sichuan University Chengdu Sichuan China; ^2^ Department of Epidemiology and Biostatistics, Institute of Systems Epidemiology, and West China‐PUMC C. C. Chen Institute of Health, West China School of Public Health and West China Fourth Hospital Sichuan University Chengdu Sichuan China; ^3^ Department of Orthopaedics, West China School of Public Health/West China Fourth Hospital Sichuan University Chengdu Sichuan China; ^4^ Department of Clinical Neuroscience Karolinska Institute Stockholm Sweden

**Keywords:** BMD, DASH diet, fracture, meta‐analysis, osteoporosis

## Abstract

This systematic review and meta‐analysis aim to investigate the association between DASH diet and risk of osteoporosis and fracture. A comprehensive literature search was conducted in PubMed, Ovid Embase, Scopus, Web of Science Core Collection and Ovid Medline databases from inception to September 2024. Two authors performed data extraction independently and assessed the risk of bias. After a systemic search, we included a total of eight eligible studies. The six cross‐sectional studies involved 17,683 individuals while the two cohort studies involved 201,062 individuals. Overall, adherence to DASH diet was not significantly associated with bone health. However, subgroup analyses revealed that adherence to DASH diet was associated with a higher level of BMD in women (β = 0.02, 95% CI = 0.02–0.03, *p* < ; $I^{2}$ = 0%; $\tau^{2}<$ 0.01) and individuals aged above 60 years (β = 0.02, 95% CI = 0.01–0.03, p < 0.01; $I^{2}$ = 61%; $\tau^{2}<$ 0.01). Large‐scale prospective cohort studies are needed to validate our findings.

**Trail registration:** PROSPERO number: CRD42024588891

## Graphical abstract

Summary of the systematic review and meta‑analysis evaluating the association between adherence to the Dietary Approaches to Stop Hypertension (DASH) diet and the risk of osteoporosis and fracture. Abbreviations: BMD, bone mineral density; DASH, dietary approaches to stop hypertension.

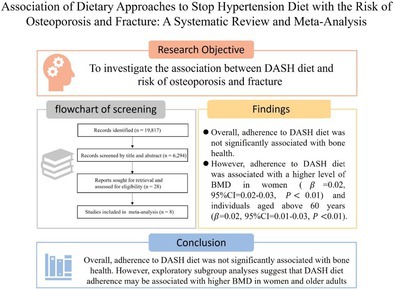

**Abbreviations**
BMD: bone mineral density
DASH: dietary approaches to stop hypertension
DXA: dual‐energy x‐ray absorptiometry
FFQ: food frequency questionnaire
NHLBI: National Heart, Lung, and Blood Institute
NOS: Newcastle‐Ottawa Scale

## 1 Introduction

Osteoporosis is an important systemic skeletal disorder characterized by reduced bone mass, deterioration of bone microarchitecture, and increased bone fragility (Rachner et al. 2011). The disease is estimated to affect approximately 200 million individuals worldwide, from which 9 million osteoporotic fractures occur annually (Shen et al. 2022). With an accelerated global aging, the incidence of osteoporosis and its related fractures is expected to rise, potentially doubling by 2050 (Clynes et al. 2020). This trend underscores a growing public health burden as the disease and its related fractures can severely impair the long‐term functioning and quality of life, eventually leading to a heightened risk of mortality (Dempster 2011). To develop effective prevention strategies, identification of etiological factors is urgently needed.

Bone mineral density (BMD) serves as an important diagnostic marker of osteoporosis and is influenced by both genetic and environmental factors (Fabiani et al. 2019). Among these factors, diet gains an increasing attention due to its modifiable nature and ease of adherence (Movassagh and Vatanparast 2017). Dietary Approaches to Stop Hypertension (DASH) diet, which emphasizes intake of whole grains, vegetables, fruits, low‐fat dairy products, legumes, and nuts (Feng et al. 2018), has been found to reduce the risk of metabolic diseases such as hypertension (Theodoridis et al. 2023), cardiovascular diseases (Jeong et al. 2023) and diabetes (Toi et al. 2020). However, the role of DASH diet in osteoporosis, a bone metabolic disorder, remains complex and multifactorial. While some evidence suggests DASH diet lacks foods rich in vitamin D, potentially leading to vitamin D deficiency and impaired calcium metabolism and bone remodeling, most studies indicate that adherence to DASH diet helps maintain bone health possibly through its anti‐inflammatory and anti‐oxidant mechanisms. Furthermore, the DASH diet has been found to be rich in potassium, calcium, vitamin C, β‐carotene and protein, all are crucial in maintaining bone health (Doyle and Cashman 2004). As a result, individuals following the DASH diet may exhibit a higher level of BMD and a lower risk of osteoporosis or fracture.

Despite a possible beneficial role of DASH diet in preventing osteoporosis, the available epidemiological evidence remains limited and inconsistent. There are only two interventional studies conducted regarding this topic. Both were originally designed to understand the effects of DASH diet on blood pressure and measured circulating bone metabolism markers as part of the secondary outcomes. One study reported DASH diet to reduce serum osteocalcin by 8%–11% and C‐terminal telopeptide of Type I collagen by 16%–18%, potentially improving bone mineral status (Lin et al. 2003). In contrast, the other study showed DASH diet to reduce serum calcitriol by 3.32 pg/mL and may be detrimental to bone health (Hassoon et al. 2018). Findings from observational studies also remain inconclusive; for example, two prospective cohort studies showed adherence to DASH diet to decrease the risk of hip fracture (Fung et al. 2018; Haring et al. 2016), but both were of limited statistical certainty. Some cross‐sectional studies reported that adherence to DASH diet may be associated with a reduced risk of osteoporosis (Shahriarpour et al. 2020; Shen et al. 2023), while another study reported DASH diet to negatively associate with BMD in adults (Zhai et al. 2023). A recent scoping review aggregated six studies to explore the association of different dietary patterns with bone health in older adults and indicated that adherence to DASH diet significantly reduced the subsequent risk of osteoporosis at the lumbar spine (HR = 0.28; 95% CI = 0.09–0.88) (Chen and Avgerinou 2023). However, this review did not focus specifically on DASH diet and included only two studies with a total sample of 8067 individuals on this topic.

Due to the inconsistency of existing results and a current lack of relevant meta‐analysis, a comprehensive review is needed to address the gaps in the literature. Therefore, this paper systematically reviews the available data on the association between the DASH diet and osteoporosis and fractures and performs a meta‐analysis.

## 2 Method

### 2.1 Protocol and Registration

The reporting of this systematic review and meta‐analysis follows the guidelines set forth by the Preferred Reporting Items for Systematic Reviews and Meta‐Analysis (PRISMA) and Meta‐analysis of Observational Studies in Epidemiology (MOOSE) checklist. To ensure transparency, we prospectively registered the detailed study protocol on the International Prospective Register of Systematic Reviews (PROSPERO; registration number: CRD42024588891).

### 2.2 Search Strategy

Two independent reviewers systematically searched PubMed, Ovid Embase, Scopus, Web of Science Core Collection, and Ovid Medline from their inception to September 2024. No language restrictions were applied. Additionally, we inspected the reference lists of pertinent reviews and articles to identify additional eligible studies. Gray literature was also consulted for potential records. All retrieved records were managed and screened using EndNote 20. Search terms encompassed “Dietary Approaches to Stop Hypertension,” “DASH,” “bone mineral density,” “BMD,” “osteoporosis” and “fracture,” and more. The complete search strategy is available in Table S1.

### 2.3 Study Selection

The following studies were included: (1) the study was published in English; (2) the study was conducted with human participants; (3) the study was an observational study using a cohort or a case–control or a cross‐sectional design; (4) the study explored the association of DASH diet on BMD or the risk of osteoporosis or fracture, which reported adjusted effect estimates (e.g., odds ratios [ORs], hazard ratios [HRs], or β‐coefficients) along with corresponding 95% confidence intervals (95% CIs).

The following studies were excluded: (1) data of the study was not available.

Two authors (B.Y. and J.H.) independently assessed study eligibility according to these predetermined inclusion and exclusion criteria. After removing duplicate records, titles and abstracts were screened to exclude clearly irrelevant records. The full texts of potentially eligible articles were retrieved and subjected to a detailed eligibility assessment. Any disagreements regarding inclusion were resolved through consensus or by adjudication from a third reviewer.

### 2.4 Data Extraction

Two authors (B.Y. and J.H.) independently performed data extraction from each included study using a standardized form. The following data were collected: first author, year of publication, study design, country, sample size, age and sex of participants, method of assessment of dietary intake, definition of DASH diet, definition of dietary adherence, method of assessment of BMD, osteoporosis and fracture, covariates included in the statistical model, and the effect estimates and their 95% CIs. In studies where multiple adjusted models were presented, the estimate from the model with the most extensive covariate adjustment was selected. All extracted data were cross‐verified for consistency and any discrepancies were resolved through iterative discussion.

### 2.5 Risk of Bias Assessment

The risk of bias of included studies was independently evaluated by two authors (B.Y. and J.H.) using the Newcastle‐Ottawa Scale (NOS) (Stang 2010) and the National Heart, Lung, and Blood Institute (NHLBI) Quality Assessment Tool for Observational Cohort and Cross‐Sectional Studies.

The NOS contains eight categories relating to methodological quality, with a total possible score of 9. Based on this score, studies are categorized as follows: low quality (0–3 points), medium quality (4–6 points), or high quality (7–9 points).

The NHLBI tool consists of 14 items each categorized as “Yes,” “No,” or “Other (cannot determine, not reported, not applicable).” These questions assessed the internal validity of each study, taking into account potential risks of bias, such as information bias, measurement bias, or outcome bias. The greater the bias (indicated by a higher number of items rated “No”), the lower the assigned rating. A rate was first calculated based on the percentage of unbiased items (items rated “Yes”) across all items, and then the overall quality of the study was assessed according to categories of rates: poor (< 50%), fair (≥50% and ≤70%), and good (>70%) (Sass et al. 2023). Any disagreement between two authors was handled by consulting a third author.

### 2.6 Statistical Analysis

We calculated summary β, OR, and HR values and 95% CIs to explore the association between DASH diet and risks of osteoporosis and fracture using a random effects model. We compared the highest adherence category with the lowest adherence category. The heterogeneity among studies (between‐study heterogeneity) was assessed using multiple approaches: the Cochran's *Q* test for statistical significance, the $I^{2}$ statistic for the magnitude, and the $\tau^{2}$ value estimated via the restricted maximum likelihood (REML) method for the between‐study variance. Consistent with conventional thresholds, $I^{2}$ values were interpreted as follows: < 25% low; 25%–50% moderate; 50%–75% high; and 75%–100% extreme heterogeneity.

To investigate potential sources of heterogeneity and examine whether the associations between the DASH diet and BMD/osteoporosis were modified by key study‐level characteristics, we conducted subgroup analyses stratified by (1) age (<60 vs. ≥ 60), (2) sex (male vs. female), and (3) ethnicity (Caucasian vs. Asian). Given the small number of studies available per subgroup, the subgroup analyses reported in this review have limited statistical power. The robustness of the primary findings was evaluated using a leave‐one‐out sensitivity analysis, sequentially removing each study to assess its influence on the pooled estimate. Given that the number of eligible studies included in each meta‐analysis was fewer than 10, formal assessment of publication bias using funnel plots or statistical tests (e.g., Begg's test) was not performed. All statistical analyses were performed using R software (version 4.3.1). *p* < 0.05 was considered statistically significant.

## 3 Results

### 3.1 Literature Search and Selection

**FIGURE 1:**
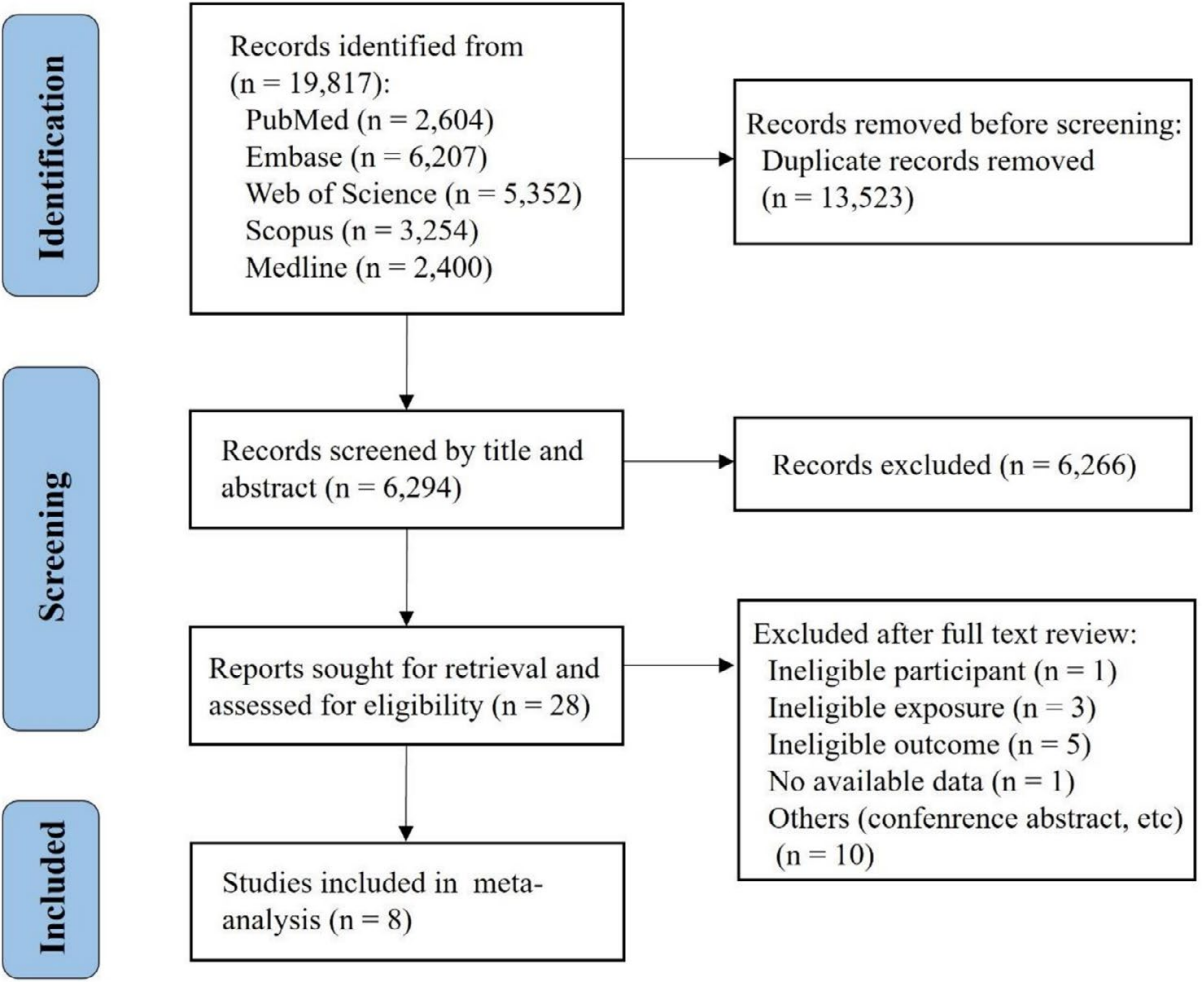
The overall flowchart of the identification, screening, and inclusion of studies.

A flowchart illustrating the literature search and selection procedure is presented in Figure 1. The initially comprehensive search identified a total of 19,817 records, which after removing duplicates left 6294 records for screening of titles and abstracts. From those records, a total of 28 reports were further retrieved for full‐texts and assessed for eligibility. A final total of 8 studies were included in our systematic review and meta‐analysis. Detailed justifications for each excluded full‐text report are summarized in Table S2.

### 3.2 Study Characteristics

The characteristics of each study are summarized in Table 1. We included eight studies (Fung et al. 2018; Haring et al. 2016; Li and Zhou 2022; Monjardino et al. 2014; Noel et al. 2020; Shahriarpour et al. 2020; Shen et al. 2023; Zhai et al. 2023) (six cross‐sectional design and two cohort design). The cross‐sectional studies involved 17,683 individuals (49.2% males; 31.2 ± 0.6 years) and the cohort studies involved 201,062 individuals (18.2% males).

**TABLE 1:** Characteristic of studies included in meta‐analysis of the DASH dietary pattern and bone mineral density/fracture.

Author	Year	Country	Design	Sample size	Male (%)	Age range	Diet assessment method	DASH dietary pattern score range/component	Definition and comparison of dietary adherence	BMD/fracture measurement	BMD measurement/fracture site	Adjustments	Quality assessment
Teresa Monjardino	2012	Portugal	Cross‐sectional study	1023	474 (46.3)	17.00 ± 0.00	FFQ	Score range: 9–45; fruit, vegetables, nuts, legumes, dairy products, whole grains, Na, red and processed meat, and sweetened beverages	A higher score indicates greater adherence to the DASH diet Tertile 3 vs. Tertile 1	DXA	Forearm BMD	Height and weight at 17 years of age, age at menarche, parental educational level, and total energy intake	Good
Sabrina E. Noel	2020	America	Cross‐sectional study	890	244 (27.4)	59.90 ± 7.60	FFQ	Score range: 8–40；fruits, vegetables, nuts and legumes, low‐fat dairy products, and whole grains, sodium, sweets and red or processed meats	A higher score indicates greater adherence to the DASH diet Quintile5 vs. Quintile1	DXA	Trochanter BMD, femoral neck BMD, total hip BMD, lumbar spine BMD	Age, sex, BMI, smoking, alcohol consumption, season of BMD measurement (fall, winter, spring, summer), osteoporosis medication use (yes/no), calcium intake (mg/day), serum vitamin D status (mg/dL), and total energy intake	Good
Z. Shahriarpor	2020	Iran	Cross‐sectional study	151	0	61.20 ± 8.20	FFQ	Score range: 8–40; fruits, vegetables, nuts and legumes, low‐fat dairy products, and whole grains, sodium, sweets and red or processed meats	A higher score indicates greater adherence to the DASH diet Quintile5 vs. Quintile1	DXA	Lumbar spine BMD	Age, BMI, smoking, education, physical activity, age at menarche, age at menopause, parity, duration of lactation, sunlight exposure, supplement intake, and energy intake	Good
Qianqian Li	2022	America	Cross‐sectional study	6294	3257 (51.7)	13.93 ± 0.07	24‐h dietary questionnaire review	Score range: 8–40; fruits, vegetables, nuts and legumes, whole grains, low‐fat dairy, sodium, red and processed meats, and sweetened beverages	A higher score indicates greater adherence to the DASH diet NR	DXA	Total femur BMD, femoral neck BMD, lumbar spine BMD	Age, sex, race, BMI, family income, serum 25‐hydroxyvitamin D, and serum cotinine	Good
Jing Shen	2023	China	Cross‐sectional study	839	324 (38.6)	61.65 ± 0.45	FFQ	Score range: 9–45; whole grains, beans, vegetables, fruits, dairy, red meat, total fat, sodium and sugar‐sweetened beverages	A higher score indicates greater adherence to the DASH diet Tertile 3 vs. Tertile 1	An ultrasonic bone densitometer	Left calcaneus BMD	Age, BMI, smoking, alcohol consumption, physical activity, age at menarche, age at menopause, hypertension, diabetes, calcium supplement intake, vitamin D supplement intake, and total energy intake	Good
Xiang Long Zhai	2023	America	Cross‐sectional study	8486	4397 (51.8)	39.07 ± 0.28	24‐h dietary questionnaire review	Score range: 0–9; total fat, saturated fat, protein, fiber, cholesterol, calcium, magnesium, sodium, and potassium	A higher score indicates greater adherence to the DASH diet Tertile 3 vs. Tertile 1	DXA	Total BMD, lumbar spine BMD, toracic spine BMD, pelvic BMD	Age, sex, race, BMI, smoking, alcohol consumption, education, ratio of family income to poverty, marital status, hypertension, and diabetes	Good
Bernhard Haring	2016	America	Cohort study	90,014	0	63.60 ± 7.40	FFQ	Score range: 8–40; fruits, vegetables, nuts and legumes, low‐fat dairy, whole grains, sodium, sweetened beverages, and red and processed meats	A higher score indicates greater adherence to the DASH diet Quintile5 vs. Quintile1	NR	Hip fractures, total fractures	Age, race, BMI, smoking, physical activity, self‐reported health, diabetes, history of fracture at 55 years or older, physical function score, number of chronic medical conditions, number of psychoactive medications, and use of hormone therapy, bisphosphonates, calcitonin, and selective estrogen receptor modulators	Good/8
Teresa T. Fung	2018	America	Cohort study	111,048	36,602 (33.0)	> 50	FFQ	Score range: 8–40; fruits, vegetables, nuts and legumes, whole grains, low‐fat dairy products, red and processed meats, sodium, and sugar‐sweetened beverages	A higher score indicates greater adherence to the DASH diet Quintile5 vs. Quintile1	Self‐reported	Hip fractures	Age, BMI, smoking, alcohol consumption, leisure‐time physical activity, postmenopausal hormone use, thiazides, Lasix, anti‐inflammatory steroids, multivitamin supplements, calcium supplement intake, vitamin D supplement intake, retinol, intake of caffeine, sugar‐sweetened beverages, diabetes, and energy intake	Good/6

Abbreviations: BMD, bone mineral density; BMI, body mass index; DASH, dietary approaches to stop hypertension; DXA, dual‐energy x‐ray absorptiometry; FFQ, Food Frequency Questionnaire; NR, not reported.

As for the cross‐sectional studies, three were conducted in the United States, two in Europe, and one in China. One study considered osteoporosis as the outcome, two studies examined BMD as the outcome, and three studies reported both BMD and osteoporosis as the outcome. For DASH diet measurements, four studies used the Food Frequency Questionnaire (FFQ) and two studies used the 24‐h dietary recall. For BMD measurements, five studies used dual‐energy x‐ray absorptiometry (DXA) and one study used the ultrasonic bone densitometer. Using the widely accepted WHO criteria for diagnosis, osteoporosis was defined as a BMD *T*‐score of ≤ −2.5.

As for the cohort studies, both were conducted in the United States with hip fracture as the outcome. The DASH diet was measured using FFQ, and fractures were derived from patient self‐report.

### 3.3 Risk of Bias Assessment

The risk of bias assessment for each included study is provided in Table 1. The NHLBI tool was used to assess the eight included studies. All literature was of relatively good quality according to our predefined categorization thresholds (“good,” 100%). Two cohort studies were further assessed using NOS. With scores of 8 and 6, both were considered of satisfactory quality (at least “moderate,” 100%).

### 3.4 Associations of DASH Diet With BMD, as Well as With the Risk of Osteoporosis or Hip Fracture

**FIGURE 2:**
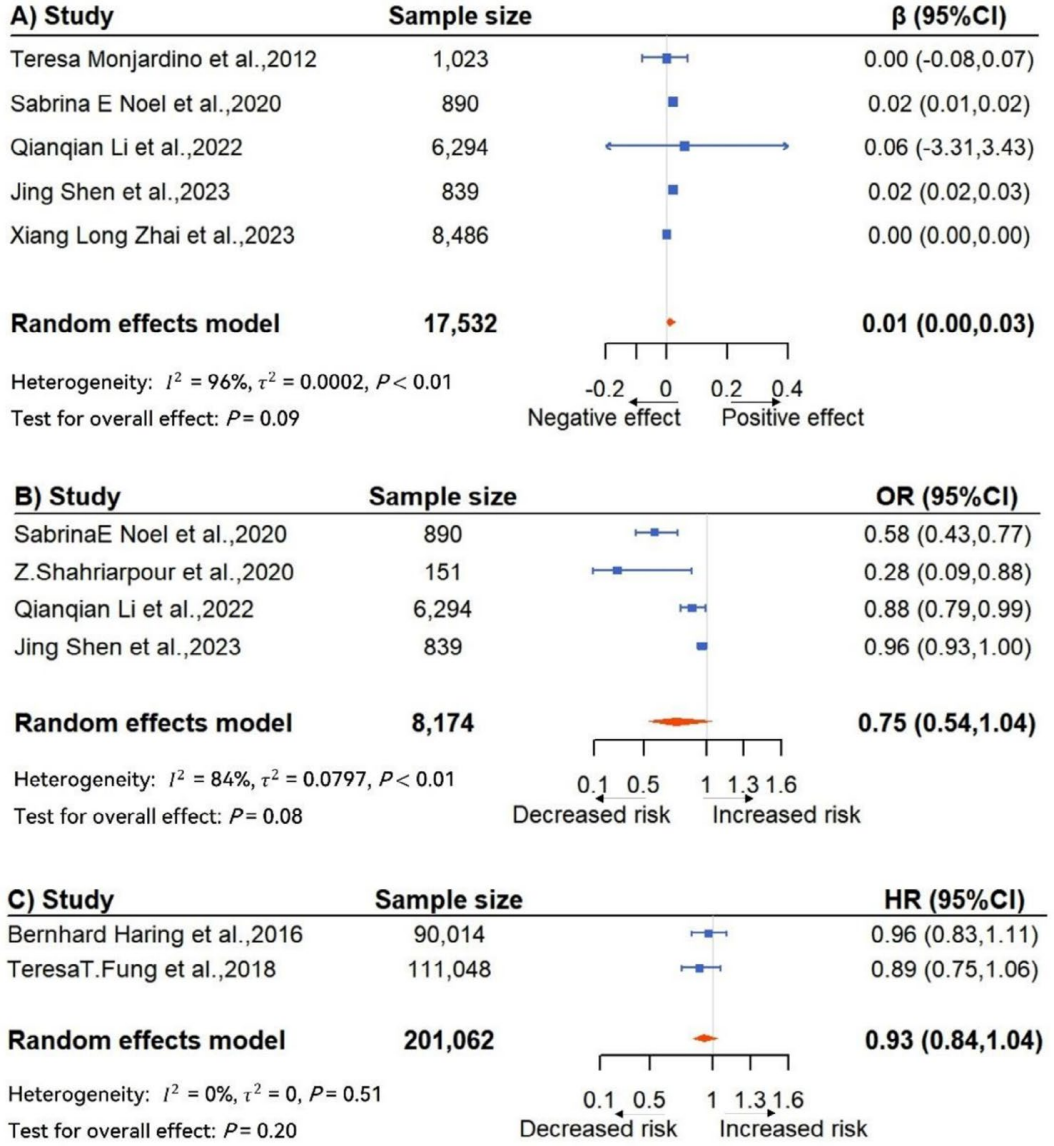
Forest plots of the association between adherence to DASH diet and (A) bone mineral density (g/cm^2^), (B) risk of osteoporosis, (C) risk of hip fracture. CI, confidence intervals; HR, hazard ratio; OR, odds ratio.

Results of the meta‐analysis are shown in Figure 2. There is no statistically significant association between adherence to the DASH diet and BMD (β = 0.01, 95% CI = 0.00–0.03, *p*= 0.09; $\tau^{2}<$ 0.01; $I^{2}$ = 96%; $p_{Q-\text{test}}$ < 0.01), the risk of osteoporosis (OR = 0.75, 95% CI = 0.54–1.04, *p* = 0.08; $\tau^{2}$= 0.08; $I^{2}$ = 84%; $p_{Q-\text{test}}$ < 0.01) or hip fracture (HR = 0.93, 95% CI = 0.84–1.04, p = 0.20; $\tau^{2}$= 0.00; $I^{2}$ = 0%; $p_{Q-\text{test}}$ = 0.51). Leave‐one‐out sensitivity analysis indicated that no single study was an outlier in terms of effect size magnitude. However, exclusion of the study using a 0–9 DASH scoring method changed the statistical significance of BMD result, highlighting the influence of scoring heterogeneity (Figures S1 and S2).

### 3.5 Subgroup Analysis

Details of the subgroup analysis are summarized in Table 2 and Figures S3–S8. Subgroup analyses of BMD showed that the heterogeneity was primarily attributed to sex and age (both $p_{\text{interaction}}$ < 0.01). Specifically, the protective effect of DASH diet on BMD was more pronounced in females (β = 0.02, 95% CI = 0.02–0.03, p < 0.01) than in males (β = 0.01, 95% CI = −0.00 to 0.02, p = 0.26); and was found only among those aged over 60 years (β = 0.02, 95% CI = 0.01–0.03, p < 0.01). Furthermore, although ethnicity was not a source of heterogeneity, adherence to DASH diet was associated with a higher BMD in the Asian population (β = 0.02, 95% CI = 0.02–0.03, p < 0.01).

**TABLE 2:** Subgroup analysis exploring the effects of DASH diet on BMD/OP in different sexes, mean age, ethnicity.

Subgroup analysis	Number of studies	Sample size	*β*/OR (95% CI)	*p*	*I* ^2^	*τ* ^2^	*p*_interaction_
Bone mineral density							
Sex							
Male	3	1042	0.01 (−0.00, 0.02)	0.26	0%	0	< 0.01
Female	3	1710	0.02 (0.02, 0.03)	< 0.01	0%	< 0.001	
Mean age, years							
< 60	3	15,803	0.00 (−0.00, 0.00)	0.11	0%	0	< 0.01
≥ 60	2	1729	0.02 (0.01, 0.03)	< 0.01	61%	< 0.001	
Ethnicity							
Asian	1	839	0.02 (0.02, 0.03)	< 0.01	NA	NA	0.07
Caucasian	4	16,693	0.01 (−0.01, 0.03)	0.43	93%	0.0002	
Risk of osteoporosis							
Sex							
Male	2	568	0.96 (0.87, 1.05)	0.33	3%	0.0014	0.38
Female	2	1161	0.74 (0.42, 1.30)	0.30	91%	0.1542	
Mean age, years							
< 60	1	6294	0.88 (0.79, 0.99)	0.03	NA	NA	0.29
≥ 60	3	1880	0.65 (0.37, 1.13)	0.13	88%	0.1847	
Ethnicity							
Asian	1	839	0.96 (0.93, 1.00)	0.07	NA	NA	0.09
Caucasian	3	7335	0.64 (0.40, 1.03)	0.07	82%	0.1216	

Abbreviations: BMD, bone mineral density; CI, confidence interval; DASH, dietary approaches to stop hypertension; NA, not available; OP, osteoporosis; OR, odds ratio.

In the subgroup analysis of osteoporosis, sex was not found to be a source of heterogeneity ($p_{\text{interaction}}$ = 0.38). The protective trend seemed to be stronger among females (OR = 0.74, 95% CI = 0.42–1.30, p = 0.30) than males (OR = 0.96, 95% CI = 0.87–1.05, p = 0.33), although both were nonsignificant. Heterogeneity among studies could not be compared by age or ethnicity as only one study included individuals younger than 60 years and only one study included the Asian population. However, given the small number of studies in each subgroup and the resulting limited statistical power, these subgroup findings should be interpreted with caution and are presented as exploratory analyses.

## 4 Discussion

To the best of our knowledge, this is the first meta‐analysis to quantify the magnitude of association between DASH diet and risks of osteoporosis and fracture, aggregating data from both cross‐sectional and cohort studies. Our analysis included eight studies with a total of 218,745 participants, indicating that overall, adherence to the DASH diet was not significantly associated with BMD, risk of osteoporosis, or hip fracture. However, subgroup analyses revealed a positive association with BMD in women and the elderly.

Our overall results are largely in line with a previous scoping review which reported a protective effect of DASH diet on BMD (Chen and Avgerinou 2023), but extend that review by performing the very first meta‐analysis that specifically quantifies the role of DASH diet on bone health encompassing measurement of BMD as well as risks of osteoporosis and fracture. While DASH diet has been widely recognized for its beneficial effects on cardiometabolic conditions—including reduced risks of cardiovascular disease (Soltani et al. 2020), diabetes (Quan et al. 2024), and hypertension (Theodoridis et al. 2023), our overall findings do not show a statistically significant association with bone health, which may be due to the limited number of studies. Nonetheless, our subgroup analyses indicate a positive association with BMD in women and the elderly. This is not surprising. A similar example is the Mediterranean diet, a diet well‐known to benefit diseases such as cardiovascular disorders (Laffond et al. 2023), hypertension (Georgoulis et al. 2024), and diabetes (Martín‐Peláez et al. 2020), which has also been demonstrated to promote bone health through the recent synthesis of evidence (Erkkilä et al. 2017; Pérez‐Rey et al. 2019).

While the overall results did not reach statistical significance, the subgroup analyses showed positive associations. For BMD, the DASH diet demonstrated a significant protective effect in females than males. This may be explained by the rapid bone loss observed in females, particularly postmenopausal women, likely due to reduced levels of free estradiol. Lower estradiol levels impair osteogenic progenitor cell activity and decrease the synthesis of collagen and bone matrix, thereby accelerating bone loss (Runolfsdottir et al. 2015; Warming et al. 2002). Similar findings have also been identified in the analysis of osteoporosis where the protective trend of DASH diet was stronger in females than males. In terms of age, the DASH diet demonstrated a significant protective effect on BMD in individuals over 60 years of age, a group characterized by accelerated bone loss and a higher prevalence of osteoporosis (Salari et al. 2021; Schuit 2006), while no such effect was observed among those under 60 years of age. Furthermore, sensitivity analysis excluding the study that used a 0–9 ranged nutrient‐based DASH score (Zhai et al. 2023) resulted in a statistically significant pooled estimate for BMD, suggesting that variation in DASH scoring methods was a major contributor to the observed heterogeneity. This finding reinforces the need for standardization of DASH adherence assessment in future studies to enable more comparable and robust meta‐analyses.

Although the exact mechanisms through which DASH diet confers protection to bone health remain unclear (Doyle and Cashman 2004), it can largely be attributed to its key components. The characteristics of DASH diet align with dietary guidelines for the prevention of osteoporosis (Weaver 2017). Higher intakes of potassium and magnesium, combined with adequate calcium, enhance dietary alkaline load, potentially reduce osteoporosis risk by inhibiting osteoclast but stimulating osteoblast activities, thereby minimizing bone calcium loss (Movassagh and Vatanparast 2017; Shariati‐Bafghi et al. 2014). Vitamin C and β‐carotene, through their antioxidant and anti‐inflammatory properties, play a vital role in maintaining the structural integrity of connective tissues and may contribute to mitigating bone loss (Thaler et al. 2022). Moreover, although the DASH diet does not specifically prioritize vitamin D intake, it is important to recognize that the main source of vitamin D is through cutaneous synthesis (Dominguez et al. 2021). Consequently, dietary vitamin D alone is unlikely to substantially attenuate the beneficial effects of the other components that DASH diet possesses on bone health.

Our results hold significant implications at both clinical and public health levels. Clinically, the DASH diet appears to attenuate bone loss, improve BMD, and reduce fracture risk, particularly among high‐risk populations such as the elderly and women (Morgan 2009). These findings provide healthcare professionals with an effective, nonpharmacological strategy for the prevention and management of osteoporosis among vulnerable individuals. From a public health perspective, the DASH diet is practical to implement and conducive to healthy aging, aligning with dietary recommendations for the prevention and control of various chronic diseases (Bojang and Manchana 2023). Results of this meta‐analysis offer robust evidence to inform public health policy development, health education initiatives, and the implementation of personalized nutritional interventions.

Several limitations should be noted. First, the sites of BMD measurement vary slightly across the literature. Although two literatures only measured forearm BMD and left heel BMD respectively, more than half of the literatures measured BMD at the lumbar spine. Therefore, lumbar spine was chosen as the primary site of analysis to guarantee power and minimize bias. Second, because most of the studies were of cross‐sectional design, our findings may only indicate association rather than causation. Third, crucial confounders related to diet and osteoporosis, including physical activity, hormone replacement therapy usage, and sun exposure, remain unassessed due to a lack of reporting in most studies. We therefore minimized such influence by incorporating results from the most fully adjusted models in each participating study. Fourth, due to the small number of studies available per subgroup, the subgroup comparisons may have been underpowered to detect true differences. Fifth, the DASH scoring methods varied across included studies, with one study using a nutrient‐based 0–9 point scale. While higher scores consistently indicated better adherence across all studies, this scoring heterogeneity may limit the comparability of pooled estimates. Sensitivity analysis excluding the 0–9 score study (Zhai et al. 2023) altered the significance of BMD result, confirming that scoring differences are an important methodological concern. Standardization of DASH scoring in future research is needed. Finally, few studies used fracture as an outcome; therefore, more literature is needed to support this relationship.

## 5 Conclusion

This systematic review and meta‐analysis of population‐based observational studies indicates that overall, adherence to the DASH diet was not significantly associated with BMD, risk of osteoporosis, or hip fracture. However, subgroup analyses revealed a positive association between DASH diet adherence and BMD in women and the elderly. These subgroup findings are exploratory, given the limited number of studies per subgroup. Our study not only bridges the gap in quantitative evidence of the DASH diet‐osteoporosis or fracture association but also suggests that the potential protective effect of the DASH diet on bone health may be more pronounced in specific populations (women and the elderly), providing a precision direction for future research. More large‐scale prospective cohort studies are needed to validate these results.

## Author Contributions

**Xiaobing Pu:** supervision, writing – review and editing. **Qin Deng:** software, validation. **Xin Song:** software, validation. **Jiaojiao Hou:** software, validation. **Bin Yang:** writing – review and editing, writing – original draft, methodology, conceptualization, software, formal analysis, data curation. **Sirui Zheng:** software, validation. **Rong Xiang:** software, validation. **Yang Qu:** software, validation. **Linna Sha:** software, validation. **Maoyao Xia:** software, validation. **Bowen Lei:** software, validation. **Jiangbo Zhu:** software, validation. **Ting Yu:** software, validation. **Ting Liu:** software, validation. **Yangdan Zhong:** software, validation. **Jinyu Zhou:** software, validation. **Xunying Zhao:** software, validation. **Tao Han:** software, validation. **Xia Jiang:** writing – review and editing, supervision.

## Funding

This work was supported by the Young Scientists Fund of the National Natural Science Foundation of China (82204170), the Science Fund for Creative Research Groups of the Science and Technology Bureau of Sichuan Province (2024NSFTD0030), and the Experimental Discipline Revitalization Plan of West China School of Public Health and West China Fourth Hospital (2023SY‐03). The funders had no role in study design, data collection, analysis, interpretation, writing of the report, or the decision for submission.

## Conflicts of Interest

The authors declare no conflicts of interest.

## Supporting information


**Figure S1:** Sensitivity analysis of DASH diet and bone mineral density (g/cm^2^).
**Figure S2:** Sensitivity analysis of DASH diet and risk of osteoporosis.
**Figure S3:** Subgroup analysis of DASH diet and bone mineral density (g/cm^2^) by sex.
**Figure S4:** Subgroup analysis of DASH diet and bone mineral density (g/cm^2^) by age.
**Figure S5:** Subgroup analysis of DASH diet and bone mineral density (g/cm^2^) by ethnicity.
**Figure S6:** Subgroup analysis of DASH diet and risk of osteoporosis by sex.
**Figure S7:** Subgroup analysis of DASH diet and risk of osteoporosis by age.
**Figure S8:** Subgroup analysis of DASH diet and risk of osteoporosis by ethnicity.
**Table S1:** Search strategy.
**Table S2:** List of studies excluded via full‐text assessment and studies included in the analyses.

## Data Availability

All relevant data are provided within the manuscript and Supporting Information. Additionally, the data analyzed for this study are available upon request from the corresponding author.
