# Supplementary material for: Association of Dietary Approaches to Stop Hypertension Diet With the Risk of Osteoporosis and Fracture: A Systematic Review and Meta‐Analysis
Source: Food Sci Nutr. 2026 May 14;14(5):e71892. doi: 10.1002/fsn3.71892 (PMC13176633; doi:10.1002/fsn3.71892)
Supplement: Supplementary file 1 — Figure S1: Sensitivity analysis of DASH diet and bone mineral density (g/cm2). Figure S2: Sensitivity analysis of DASH diet and risk of osteoporosis. Figure S3: Subgroup analysis of DASH diet and bone mineral density (g/cm2) by sex. Figure S4: Subgroup analysis of DASH diet and bone mineral density (g/cm2) by age. Figure S5: Subgroup analysis of DASH diet and bone mineral density (g/cm2) by ethnicity. Figure S6: Subgroup analysis of DASH diet and risk of osteoporosis by sex. Figure S7: Subgroup analysis of DASH diet and risk of osteoporosis by age. Figure S8: Subgroup analysis of DASH diet and risk of osteoporosis by ethnicity. Table S1: Search strategy. Table S2: List of studies excluded via full‐text assessment and studies included in the analyses. [file FSN3-14-e71892-s001.docx]

**Table S1. Search strategy**

**PubMed** (September 3, 2024)

| **No.** | **Search terms** | **Result** |
| --- | --- | --- |
| 1 | "Dietary Approaches to Stop Hypertension"[MeSH Terms] | 489 |
| 2 | "dietary approaches to stop hypertension"[Title/Abstract] OR "DASH"[Title/Abstract] | 10,782 |
| 3 | "DASH"[Title/Abstract] | 10,552 |
| 4 | "diet*"[Title/Abstract] OR "score*"[Title/Abstract] OR "assess*"[Title/Abstract] OR "scale*"[Title/Abstract] OR "index"[Title/Abstract] OR "indices"[Title/Abstract] | 6,813,145 |
| 5 | 3 AND 4 | 7,485 |
| 6 | 1 OR 2 OR 5 | 10,820 |
| 7 | "osteoporosis, postmenopausal"[MeSH Terms] OR "osteoporosis"[MeSH Terms] OR "fractures, bone"[MeSH Terms] | 263,374 |
| 8 | "bone mineral density"[Title/Abstract] OR "BMD"[Title/Abstract] OR "bone mass density"[Title/Abstract] OR "fracture*"[Title/Abstract] OR "osteoporo*"[Title/Abstract] OR "bone*"[Title/Abstract] | 1,139,608 |
| 9 | 7 OR 8 | 1,176,931 |
| 10 | 6 AND 9 | 2,604 |

**Embase** (via Ovid) (September 3, 2024)

| **No.** | **Search terms** | **Result** |
| --- | --- | --- |
| 1 | Dietary Approaches to Stop Hypertension.de. | 611 |
| 2 | dietary approaches to stop hypertension.ab. or dietary approaches to stop hypertension.at. or DASH.ab. or DASH.at. | 32,498 |
| 3 | DASH.ab. or DASH.at. | 31,714 |
| 4 | diet*.at. or diet*.ab. or score*.at. or score*.ab. or assess*.at. or assess*.ab. or scale*.at. or scale*.ab. or index.at. or index.ab. or indices.at. or indices.ab. | 22,622,852 |
| 5 | 3 AND 4 | 24,140 |
| 6 | 1 OR 2 OR 5 | 32,558 |
| 7 | osteoporosis.de. | 204,591 |
| 8 | fractures.de. | 1,074 |
| 9 | bone mineral density.at. or bone mineral density.ab. or BMD.at. or BMD.ab. or bone mass density.at. or bone mass density.ab. or fracture*.at. or fracture*.ab. or osteoporo*.at. or osteoporo*.ab. or bone*.at. or bone*.ab. | 3,260,141 |
| 10 | 7 OR 8 OR 9 | 3,317,271 |
| 11 | 6 AND 10 | 7,339 |
| 12 | Limits to humans and English | 6,207 |

**Web of Science** **Core Collection** (September 3, 2024)

| **No.** | **Search terms** | **Result** |
| --- | --- | --- |
| 1 | "Dietary Approaches to Stop Hypertension"[MeSH Terms] |  |
| 2 | ((TS=(Dietary Approaches To Stop Hypertension)) OR TS=(dietary approaches to stop hypertension)) OR TS=(DASH) | 75,929 |
| 3 | TS=(DASH) | 75,557 |
| 4 | (((((TS=(diet*)) OR TS=(score*)) OR TS=(assess*)) OR TS=(scale*)) OR TS=(index)) OR TS=(indices) | 22,282,693 |
| 5 | 3 AND 4 | 20,812 |
| 6 | 1 OR 2 OR 5 | 75,929 |
| 7 | (TS=(osteoporosis)) OR TS=(fractures) | 1,696,315 |
| 8 | (((((TS=(bone mineral density)) OR TS=(BMD)) OR TS=(bone mass density)) OR TS=(fracture*)) OR TS=(osteoporo*)) OR TS=(bone*) | 4,647,446 |
| 9 | 7 OR 8 | 4,676,917 |
| 10 | 6 AND 9 | 5,352 |

**Scopus** (September 3, 2024)

| **No.** | **Search terms** | **Result** |
| --- | --- | --- |
| 1 | "Dietary Approaches to Stop Hypertension"[MeSH Terms] |  |
| 2 | (TITLE-ABS-KEY(dietary approaches to stop hypertension) OR TITLE-ABS-KEY(Dietary Approaches To Stop Hypertension) OR TITLE-ABS-KEY(DASH)) | 16,485 |
| 3 | TITLE-ABS-KEY(DASH) | 16,275 |
| 4 | (TITLE-ABS-KEY(diet*) OR TITLE-ABS-KEY(score*) OR TITLE-ABS-KEY(assess*) OR TITLE-ABS-KEY(scale*) OR TITLE-ABS-KEY(index) OR TITLE-ABS-KEY(indices)) | 16,235,672 |
| 5 | 3 AND 4  ( ( TITLE-ABS-KEY ( diet* ) OR TITLE-ABS-KEY ( score* ) OR TITLE-ABS-KEY ( assess* ) OR TITLE-ABS-KEY ( scale* ) OR TITLE-ABS-KEY ( index ) OR TITLE-ABS-KEY ( indices ) ) AND ( TITLE-ABS-KEY ( dash ) ) ) | 9,615 |
| 6 | 1 OR 2 OR 5  ( ( ( TITLE-ABS-KEY ( diet* ) OR TITLE-ABS-KEY ( score* ) OR TITLE-ABS-KEY ( assess* ) OR TITLE-ABS-KEY ( scale* ) OR TITLE-ABS-KEY ( index ) OR TITLE-ABS-KEY ( indices ) ) AND ( TITLE-ABS-KEY ( dash ) ) ) OR ( TITLE-ABS-KEY ( dietary AND approaches AND to AND stop AND hypertension ) OR TITLE-ABS-KEY ( dietary AND approaches AND to AND stop AND hypertension ) OR TITLE-ABS-KEY ( dash ) ) ) | 16,485 |
| 7 | ( TITLE-ABS-KEY ( osteoporosis ) OR TITLE-ABS-KEY ( fractures ) ) | 1,110,112 |
| 8 | ( TITLE-ABS-KEY ( bone AND mineral AND density ) OR TITLE-ABS-KEY ( BMD ) OR TITLE-ABS-KEY ( bone AND mass AND density ) OR TITLE-ABS-KEY ( fracture* ) OR TITLE-ABS-KEY ( osteoporo* ) OR TITLE-ABS-KEY ( bone* ) ) | 2,551,213 |
| 9 | 7 OR 8  ( ( TITLE-ABS-KEY ( osteoporosis ) OR TITLE-ABS-KEY ( fractures ) ) OR ( TITLE-ABS-KEY ( bone AND mineral AND density ) OR TITLE-ABS-KEY ( BMD ) OR TITLE-ABS-KEY ( bone AND mass AND density ) OR TITLE-ABS-KEY ( fracture* ) OR TITLE-ABS-KEY ( osteoporo* ) OR TITLE-ABS-KEY ( bone* ) ) ) | 2,551,213 |
| 10 | 6 AND 9  ( ( ( ( TITLE-ABS-KEY ( diet* ) OR TITLE-ABS-KEY ( score* ) OR TITLE-ABS-KEY ( assess* ) OR TITLE-ABS-KEY ( scale* ) OR TITLE-ABS-KEY ( index ) OR TITLE-ABS-KEY ( indices ) ) AND ( TITLE-ABS-KEY ( dash ) ) ) OR ( TITLE-ABS-KEY ( dietary AND approaches AND to AND stop AND hypertension ) OR TITLE-ABS-KEY ( dietary AND approaches AND to AND stop AND hypertension ) OR TITLE-ABS-KEY ( dash ) ) ) AND ( ( TITLE-ABS-KEY ( osteoporosis ) OR TITLE-ABS-KEY ( fractures ) ) OR ( TITLE-ABS-KEY ( bone AND mineral AND density ) OR TITLE-ABS-KEY ( BMD ) OR TITLE-ABS-KEY ( bone AND mass AND density ) OR TITLE-ABS-KEY ( fracture* ) OR TITLE-ABS-KEY ( osteoporo* ) OR TITLE-ABS-KEY ( bone* ) ) ) ) | 3,254 |

**Medline** (September 3, 2024)

| **No.** | **Search terms** | **Result** |
| --- | --- | --- |
| 1 | MH Dietary Approaches to Stop Hypertension | 478 |
| 2 | AB dietary approaches to stop hypertension OR TI dietary approaches to stop hypertension OR AB DASH OR TI DASH | 10,613 |
| 3 | AB DASH OR TI DASH | 10,353 |
| 4 | AB diet* OR TI diet* OR AB score* OR TI score* OR AB assess* OR TI assess* OR AB scale* OR TI scale* OR AB index OR TI index OR AB indices OR TI indices | 6,671,016 |
| 5 | 3 AND 4 | 7,289 |
| 6 | 1 OR 2 OR 5 | 7,289 |
| 7 | MH osteoporosis OR MH fractures | 52,367 |
| 8 | AB bone mineral density OR TI bone mineral density OR AB BMD OR TI BMD OR AB bone mass density OR TI bone mass density OR AB fracture* OR TI fracture* OR AB osteoporo* OR TI osteoporo* OR AB bone* OR TI bone* | 1,104,871 |
| 9 | 7 OR 8 | 1,104,871 |
| 10 | 6 AND 9 | 2,400 |

**Table S2. List of studies excluded via full-text assessment and studies included in the analyses.**

| 1.Ineligible participant (n = 1) ^[1]^ |
| --- |
| 2.Ineligible exposure (n = 3) ^[2-8]^ |
| 3.Ineligible outcome (n = 5) ^[9-15]^ |
| 4.No available data (n = 1) ^[16-20]^ |

1. Doyle L, Cashman KD. The effect of nutrient profiles of the Dietary Approaches to Stop Hypertension (DASH) diets on blood pressure and bone metabolism and composition in normotensive and hypertensive rats. Br J Nutr. 2003 May;89(5):713-24.

2. Lanham-New SA. The balance of bone health: tipping the scales in favor of potassium-rich, bicarbonate-rich foods. J Nutr. 2008 Jan;138(1):172S-177S.

3. McTiernan A, Wactawski-Wende J, Wu L, Rodabough RJ, Watts NB, Tylavsky F, Freeman R, Hendrix S, Jackson R; Women's Health Initiative Investigators. Low-fat, increased fruit, vegetable, and grain dietary pattern, fractures, and bone mineral density: the Women's Health Initiative Dietary Modification Trial. Am J Clin Nutr. 2009 Jun;89(6):1864-76.

4. Woo J, Kwok T, Leung J, Tang N. Dietary intake, blood pressure and osteoporosis. J Hum Hypertens. 2009 Jul;23(7):451-5.

5. Hassoon, A., et al. (2015). "Effects of dietary patterns on vitamin D and other markers of bone-mineral metabolism: Results from the DASH trial." Circulation 131(SUPPL. 1).

6. New SA. Intake of fruit and vegetables: implications for bone health. Proc Nutr Soc. 2003 Nov;62(4):889-99.

7. Whitting, S. J. and H. Vatanparast. "Nutritional interventions in osteoporosis." Geriatrics and Aging 2005 Oct;8(9): 14-20.

8. McTiernan A, Wactawski-Wende J, Wu L, Rodabough RJ, Watts NB, Tylavsky F, Freeman R, Hendrix S, Jackson R; Women's Health Initiative Investigators. Low-fat, increased fruit, vegetable, and grain dietary pattern, fractures, and bone mineral density: the Women's Health Initiative Dietary Modification Trial. Am J Clin Nutr. 2009 Jun;89(6):1864-76.

9. Lin PH, Ginty F, Appel LJ, Aickin M, Bohannon A, Garnero P, Barclay D, Svetkey LP. The DASH diet and sodium reduction improve markers of bone turnover and calcium metabolism in adults. J Nutr. 2003 Oct;133(10):3130-6.

10. Nowson CA, Patchett A, Wattanapenpaiboon N. The effects of a low-sodium base-producing diet including red meat compared with a high-carbohydrate, low-fat diet on bone turnover markers in women aged 45-75 years. Br J Nutr. 2009 Oct;102(8):1161-70.

11. Hassoon A, Michos ED, Miller ER, Crisp Z, Appel LJ. Effects of Different Dietary Interventions on Calcitriol, Parathyroid Hormone, Calcium, and Phosphorus: Results from the DASH Trial. Nutrients. 2018 Mar 17;10(3):367.

12. Champagne CM. Dietary interventions on blood pressure: the Dietary Approaches to Stop Hypertension (DASH) trials. Nutr Rev. 2006 Feb;64(2 Pt 2):S53-6.

13. Zhang Y, Francis EC, Xia T, Kemper K, Williams J, Chen L. Adherence to DASH dietary pattern is inversely associated with osteoarthritis in Americans. Int J Food Sci Nutr. 2020 Sep;71(6):750-756.

14. Doyle L, Cashman KD. The DASH diet may have beneficial effects on bone health. Nutr Rev. 2004 May;62(5):215-20.

15. Mirzaei, K. H., et al. (2018). "Evaluation of the relationship between major dietary patterns with bone loss rate and bone biomarkers in postmenopausalwomen." Osteoporosis International 29(1 Supplement 1): S361.

16. Doyle, L. (2004). "Whole diets, nutrients and bioactive food components as determinants of bone health and blood pressure."

17. Chen H, Avgerinou C. Association of Alternative Dietary Patterns with Osteoporosis and Fracture Risk in Older People: A Scoping Review. Nutrients. 2023 Oct 3;15(19):4255. （重复）

18. Wengreen, H. J., et al. (2009). "DASH-STYLE DIET PATTERN AND RISK OF OSTEOPOROTIC HIP FRACTURE IN ELDERLY RESIDENTS OF UTAH." American Journal of Epidemiology 169: S93-S93.

19. Noel, S., et al. (2019). "The dietary approaches to stop hypertension (DASH) index is associated with a lower odds of osteoporosis in adults aged 50 y and older." Journal of Bone and Mineral Research 34(Supplement 1): 351.

20. Lin, Y.-C. and J.-Y. Lin (2020). "Correlations between dietary patterns of Mediterranean-type and Dietary Approaches to Stop Hypertension style and bone health status in adults." Journal of Bone and Mineral Research 35: 232-232.


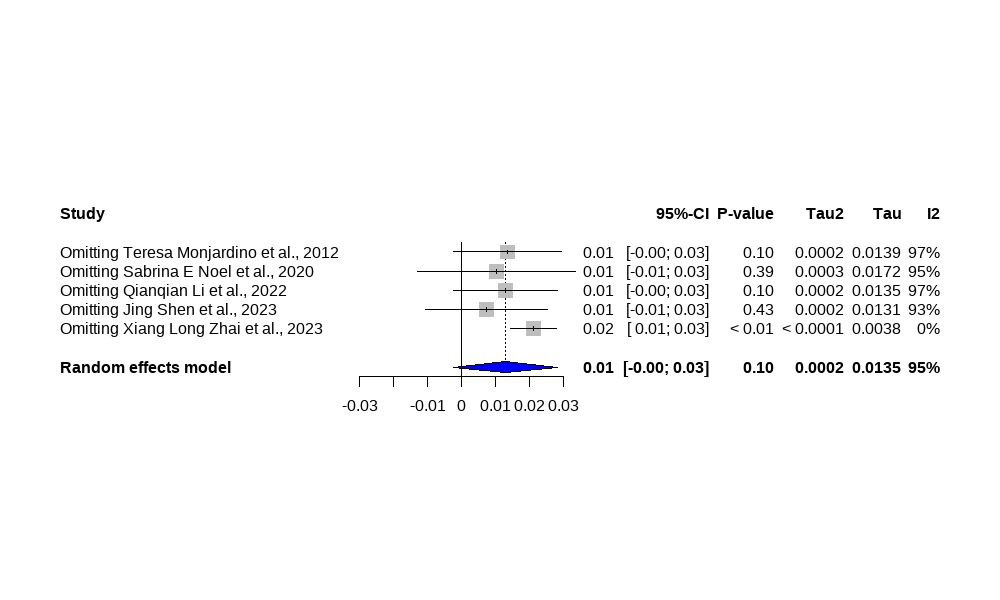


**Figure S1.** [Sensitivity analysis](javascript:;) of DASH diet and bone mineral density (g/cm2).


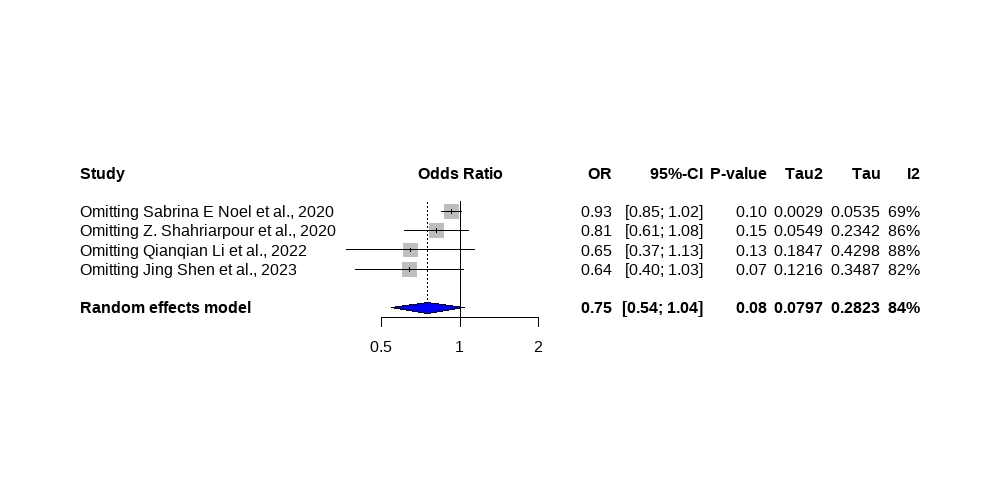


**Figure S2.** [Sensitivity analysis](javascript:;) of DASH diet and risk of osteoporosis.


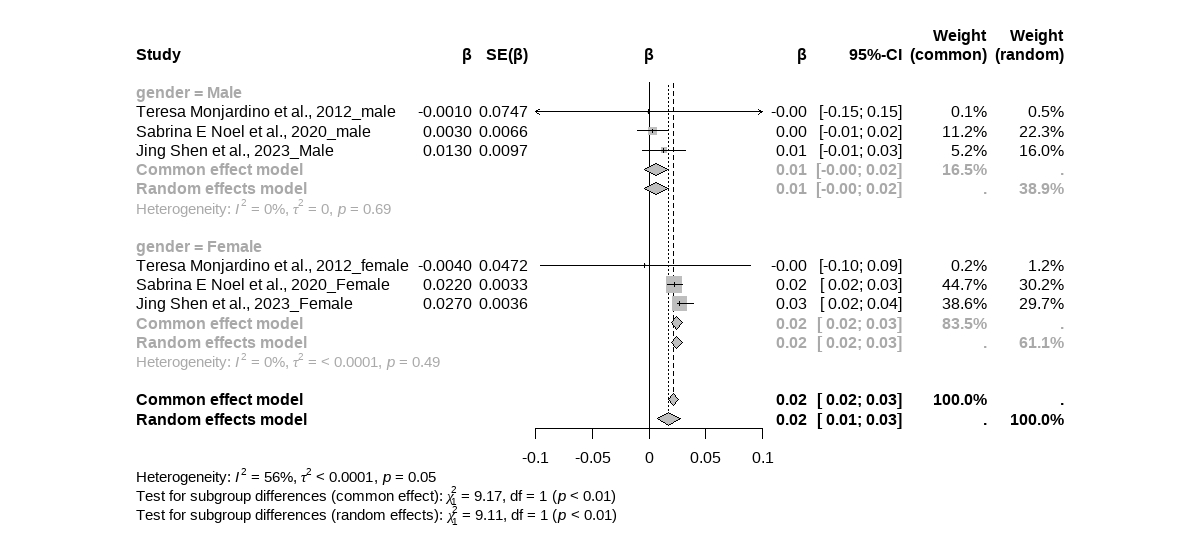


**Figure S3.** [Subgroup analysis](javascript:;) of DASH diet and bone mineral density (g/cm^2^) by sex.


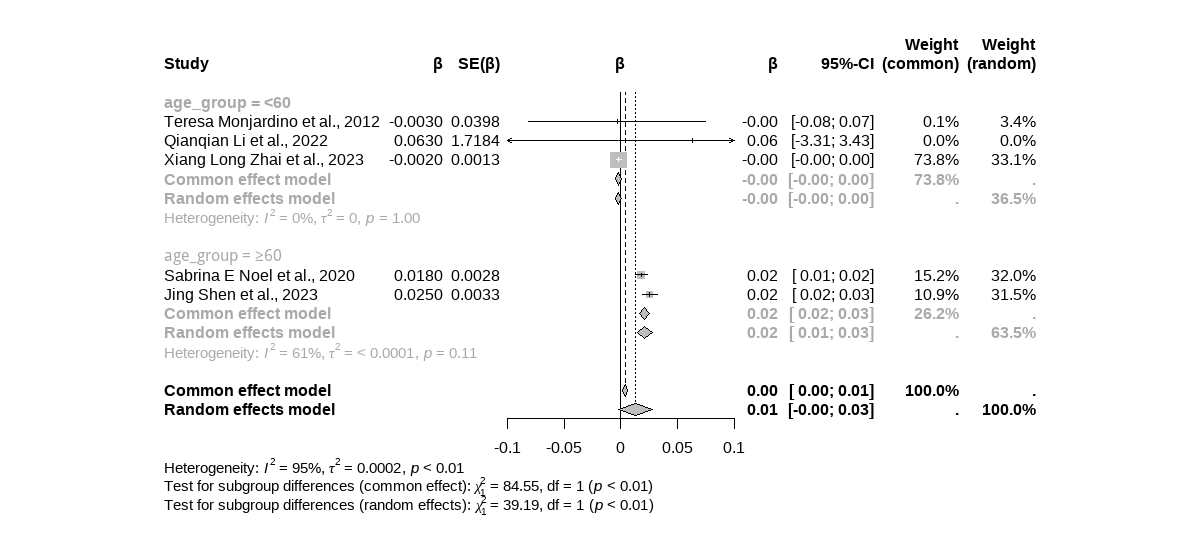


**Figure S4.** [Subgroup analysis](javascript:;) of DASH diet and bone mineral density (g/cm^2^) by age.


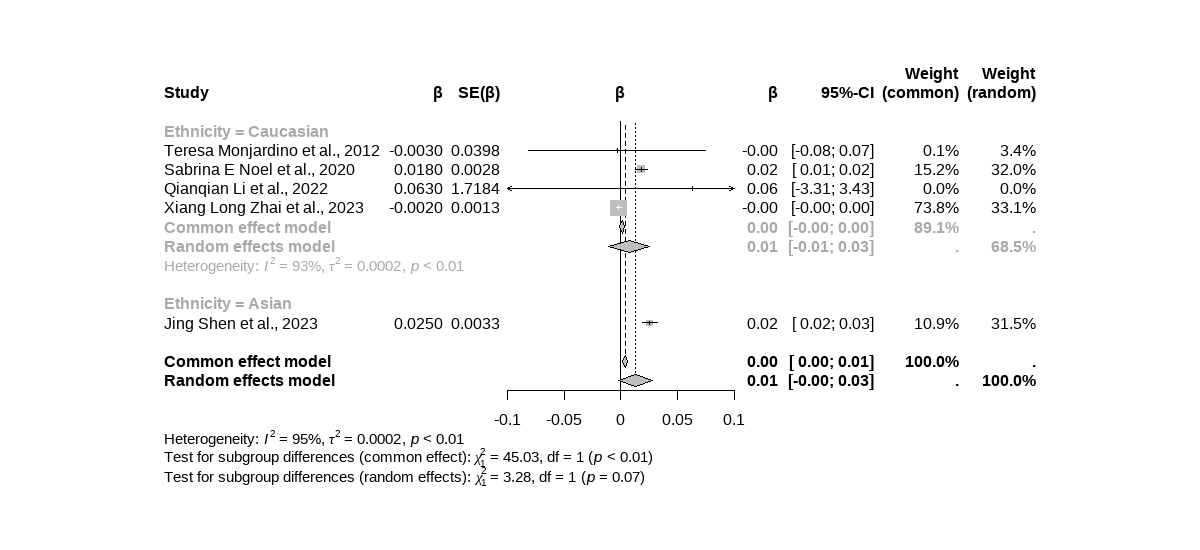


**Figure S5.** [Subgroup analysis](javascript:;) of DASH diet and bone mineral density (g/cm^2^) by ethnicity.


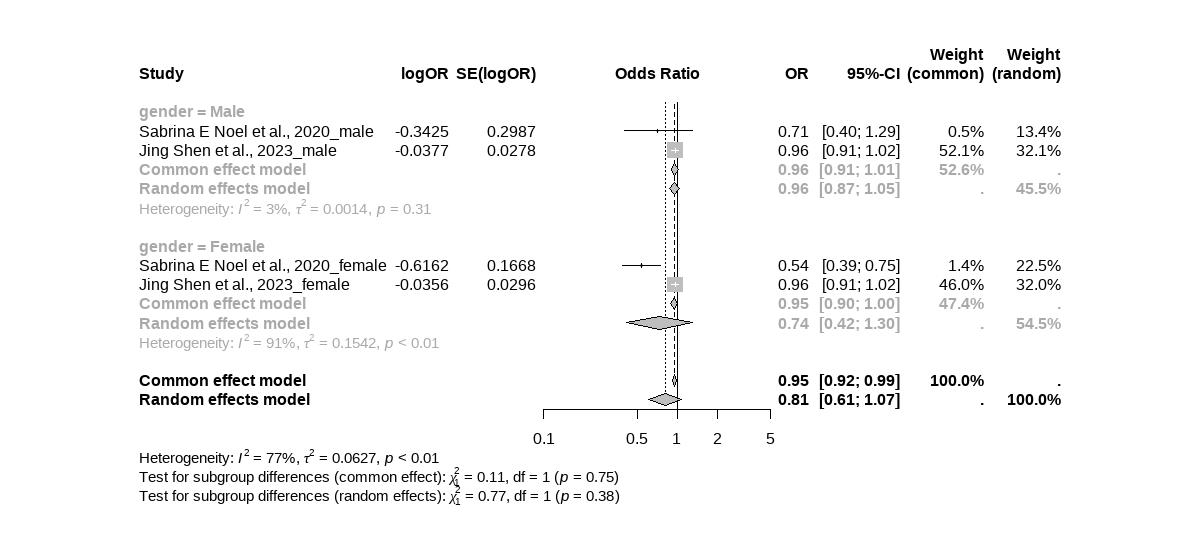


**Figure S6.** [Subgroup analysis](javascript:;) of DASH diet and risk of osteoporosis by sex.


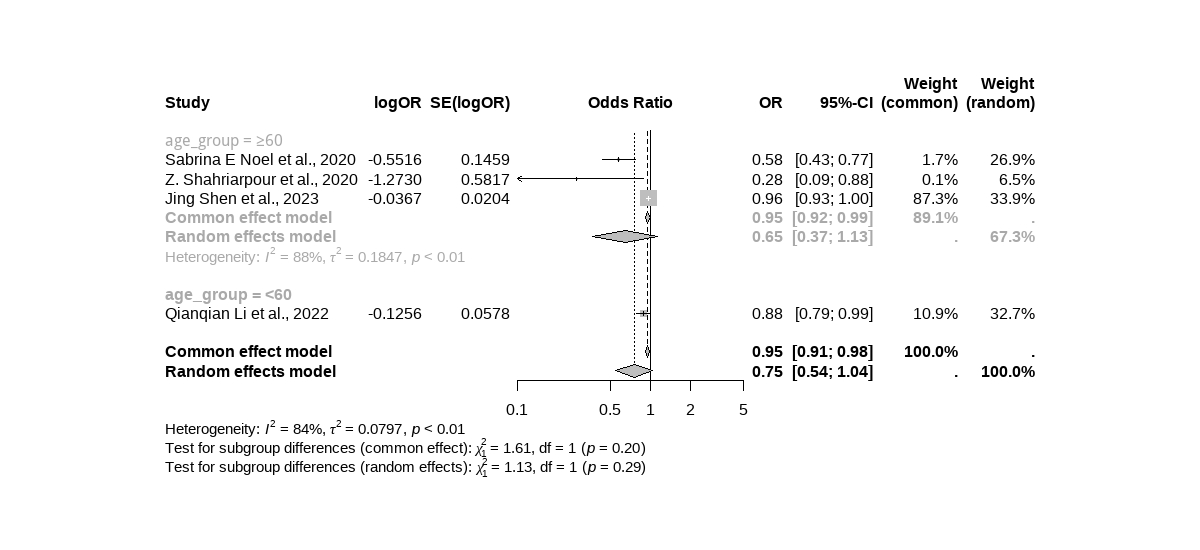


**Figure S7.** [Subgroup analysis](javascript:;) of DASH diet and risk of osteoporosis by age.


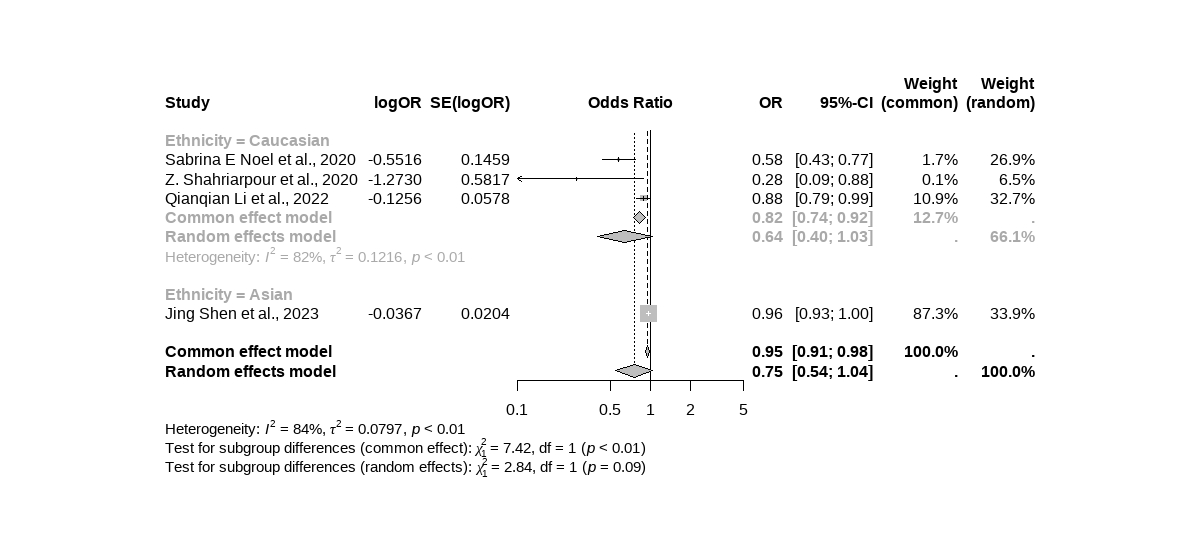


**Figure S8.** [Subgroup analysis](javascript:;) of DASH diet and risk of osteoporosis by ethnicity.
